# Spatially explicit assessment of genetic variation to inform conservation effort for an endangered Mediterranean conifer, *Cedrus atlantica*

**DOI:** 10.1002/ece3.9613

**Published:** 2022-12-12

**Authors:** Javier Bobo‐Pinilla, Diego Nieto Lugilde, Anass Terrab, Francisco Balao, Julio Peñas

**Affiliations:** ^1^ Department of Botany and Plant Physiology University of Salamanca Salamanca Spain; ^2^ Biobanco de ADN Vegetal, Edificio Multiusos I+D+i Salamanca Spain; ^3^ Departmento de Botánica, Ecología y Fisiología Vegetal Universidad de Córdoba Córdoba Spain; ^4^ Departamento de Biología Vegetal y Ecología, Facultad de Biología Universidad de Sevilla Sevilla Spain; ^5^ Department of Botany University of Granada Granada Spain

**Keywords:** atlas cedar, bioclimatic variables, genetic diversity and rarity, gradient forest model, in situ‐ex situ conservation

## Abstract

Preserving the genetic diversity of forest species is critical for maintaining their adaptive potential and allowing for generation turnover in forest ecosystems. Considering an uncertain future, it is necessary to establish reliable genetic conservation strategies to optimize the genetic variation preserved within populations in a spatially explicit context to assist decision‐makers. Hence, we aimed to incorporate genetic information into spatially designed conservation actions. *Cedrus atlantica* is a large, long‐lived conifer native to the mountains of North Africa, threatened by extinction. The relevant genetic units for conservation were selected using Bayesian analysis. The relative contribution of the populations to the genetic pool that maximized the species' genetic diversity was calculated to design an optimal seed bank. Finally, the relationship between the genetic composition and bioclimatic variables was estimated and projected throughout the study area under current and future climatic conditions. Three relevant genetic units were found for *C. atlantica* conservation that maximizes genetic diversity in a spatial context. Bioclimatic variables with the highest influence on genetic composition were closely related to climate warming and decreased soil water availability. We identified the role of genetic markers in designing a reliable conservation strategy for forest trees considering climate change, increased deforestation, and aridity. Projections of genetic composition due to the climate in the study region of North Africa provide spatially explicit guidance for optimizing the selection and preservation of seed banks.

## Graphical abstract

This paper aims to establish a reliable conservation proposal, both ex situ and in situ, regarding the genetic pattern of *Cedrus atlantica* populations and the bioclimatic conditions of the areas where it inhabits. The proposal includes the most appropriate areas to consider in order to preserve the values of genetic diversity and rarity, three seed banks to be designed looking to maximize the genetic diversity of reinforcements and the climatic areas in which the different seed banks should be used.

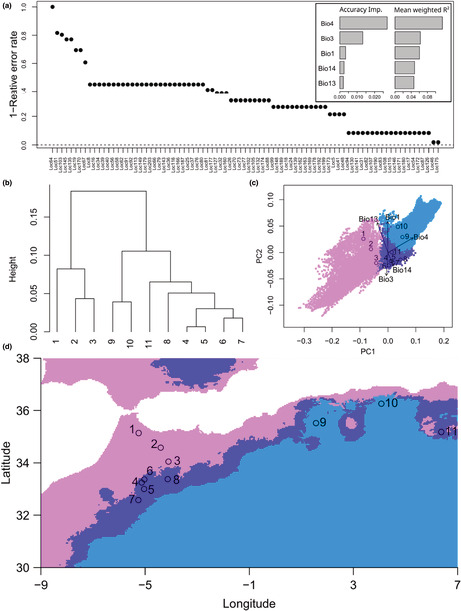

## 1 INTRODUCTION

Anticipating biodiversity changes and avoiding massive loss of species are crucial environmental challenges of the Anthropocene, as human actions are heating the planet alarmingly (Sala et al., 2000; Wardle et al., 2011). Among potential corrective actions, reducing carbon emissions and sequestering carbon by preserving and restoring ecosystems are entirely possible (Pimm, 2021). However, conservation and restoration initiatives must be interdisciplinary, with broad and complementary scopes at multiple levels, from communities and ecosystems to species or genetic compositions (Young & Clarke, 2000). Therefore, preserving genetic diversity within species and implementing strategies to prevent their genetic erosion (Laikre et al., 2020) require focusing on the genetic distinctiveness of populations (Bobo‐Pinilla et al., 2022). However, for most species, such information and studies are lacking.

Maintaining the genetic diversity of trees (such as forest genetic resources) is relevant due to their role in adaptation and since they allow generation turnover in forest ecosystems (Lefèvre et al., 2013), which promotes global biodiversity and ecological resilience (Jensen & Svenning, 2021). There is greater attention to the genetic conservation of populations when designing management and conservation plans for rare and/or threatened species. Despite their biodiversity, compared with Central European forests, Mediterranean forests are delicate ecosystems whose current conservation status is due to natural and anthropogenic drivers (Quézel et al., 1999). For example, Mediterranean vegetation has experienced distribution changes during glacial–interglacial cycles (Svenning et al., 2008) and historical deforestation of vast areas for agronomic use (Mendoza‐Fernández et al., 2021), which has reduced the distribution range and genetic diversity of the species. Among Mediterranean forests, the biodiversity and ecological importance of conifer forests (such as *Abies pinsapo* Boiss., *A. marocana* Trab., *A. numidica* Carr., *A. nebrodensis* (Lojac.) Mattei, and *Cupressus atlantica* Gaussen, or *C. sempervirens* L.), combined with their narrow distribution, have made them the focus of Mediterranean conservation research (Rundel, 2019).

Atlas cedar (*Cedrus atlantica* (Endl.) Manetti ex Carrière), a conifer tree endemic to the mountain ranges of Morocco and Algeria, is an endangered species (IUCN, 2017) and an appropriate study case for conservation research. In Morocco, *C. atlantica* occurs in two separated geographical areas: the Rif Mountains (160 km^2^) and the Middle and Eastern High Atlas (1160 km^2^), whereas in Algeria, it occurs in distant areas and covers ~300 km^2^ in the Tell Atlas and Aurès Mountains. However, the total area of occupancy (1300–1500 km^2^) is estimated to be a small fraction of its extent of occurrence (20,000 km^2^; Linares et al., 2011; Terrab et al., 2008; Thomas, 2013) due to complex interactions between multiple drivers of natural and anthropogenic origin. Their presence in NW Africa has been documented since the Messinian period (ca. 7–5 Ma; Feddi et al., 2011; Magri, 2012). During the Roman Empire, the use of forest resources intensified. Mining began in mountainous areas (Cheddadi et al., 2019), but *C. atlantica* forests remained intact at that time (Cheddadi et al., 2015). The expansion of the Arab Empire meant an increase in forest exploitation, although metallurgical industries did not continue (Cheddadi et al., 2015). Anthropogenic pressure continued to exist owing to grazing and logging and complemented threats, such as extreme climatic variations, by increasing temperatures and decreasing precipitation (Cheddadi et al., 2017). Consequently, Morocco and Algeria lost ~75% of their original *C. atlantica* forests between 1940 and 1982 (Fennane & Benabid, 1994).

Previous studies have examined the genetic characteristics, diversity, and structure of *C. atlantica*, shedding light on its conservation status and biogeographical history. For instance, similar to other *Cedrus* species, *C. atlantica* has a diploid chromosome number of 2n = 2x = 24, with no polyploids described thus far. The average genome size is 15.7 × 10^9^ base pairs per C (Bou Dagher‐Kharrat et al., 2001). This shows a relatively high within‐population genetic diversity at present (Terrab et al., 2008), although some populations have extremely low diversity. Moreover, it appears that the Rif and Middle Atlas populations were genetically isolated in the past, and the gene flow between them has become possible recently (Terrab et al., 2006). Two genetic groups of populations were revealed by nonmetric multidimensional scaling (NMDS) analysis of the *F*_ST_ matrix by Terrab et al. (2008). A population group consisting of the Rif *C. atlantica* forests and part of the Middle Atlas and another of the Algerian and the remaining Middle Atlas *C. atlantica* forests. The latter exhibited higher genetic diversity, suggesting that *C. atlantica* populations could persist during the Last Glacial Maximum in the RIF areas and subsequently expand in this region. To our knowledge, no previous study has used this information to design specific conservation actions (such as identifying relevant genetic groups for conservation or designing seed bank samplings that optimize preserving genetic diversity).

Given the great threat to *C. atlantica* natural populations from climatic and anthropic pressures, it appears necessary to establish robust conservation measures for the species. This must be based on the genetic composition and structure of the remaining populations and on spatial models to investigate the potential relationship between genetic structure and climate, allowing the estimation of the most suitable areas for different genetic groups. These advances are necessary to set in situ and ex situ conservation measures: prioritizing the populations to conserve, guiding the creation of seed banks adequately representing the species' genetic diversity, and using these seed banks to ensure a correct match between genetic diversity and climatic suitability.

To establish a reliable conservation strategy for *C. atlantica* in a spatial context assisting decision‐makers, we aimed to: (a) select the genetic populations for an optimal in situ management plan, (b) calculate the genetic contribution of populations to generate a seed bank that optimizes diversity values for ex situ conservation, and (c) model genetic structure as a function of environmental conditions to guide in situ and ex situ actions (defined in the previous objectives), providing spatially explicit guidance for conservation and management plans.

## 2 MATERIAL AND METHODS

We analyzed current population genetic data for *C. atlantica*, selecting relevant genetic units for conservation (RGUCs) that maximized genetic diversity through its extant populations (in situ conservation). Based on this data, we designed a seed sampling strategy to optimize the selection and preservation of its genetic diversity (ex situ conservation). In addition, we studied the relationship between the genetic structure of the populations and climatic variables. Relating the complete genetic composition at the population level with multiple climatic variables simultaneously, we made genetic composition projections due to the climate in the study region.

### 2.1 Population sampling and genetic information

**FIGURE 1:**
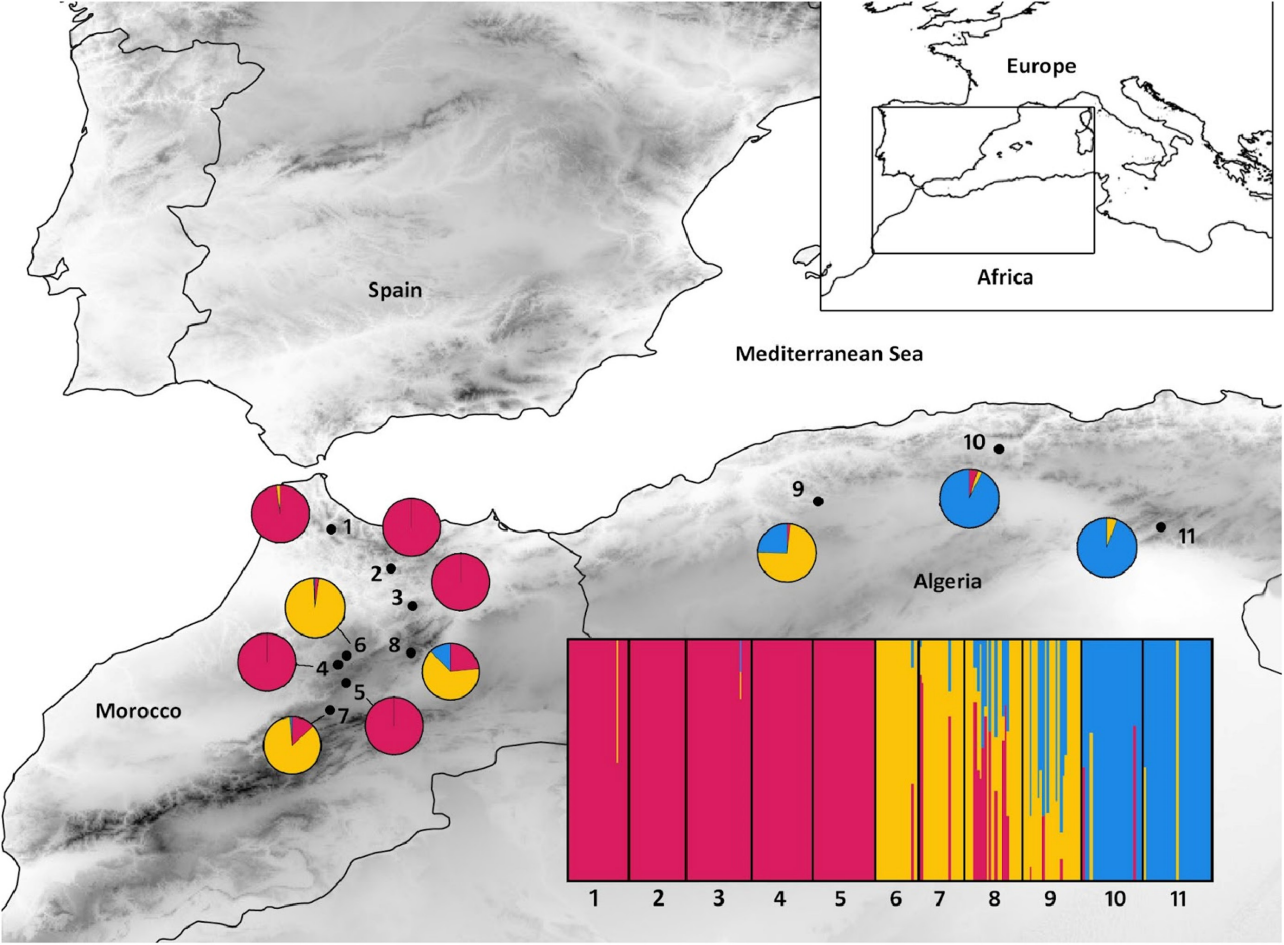
Distribution of the Bayesian admixture results obtained with Bayesian analysis of population structure software for *Cedrus atlantica*. Different colors represent the different genetic clusters (i.e., C1 = red, C2 = orange, C3 = blue).

The amplified fragment length polymorphism (AFLP) data set of *C. atlantica* from Terrab et al. (2008) was used to characterize genetic diversity. We briefly present the methods used to obtain the AFLP data set of 255 samples (trees) from 11 populations covering the entire range of this species (Morocco and Algeria; Table 1 and Figure 1). Genomic DNA was extracted using the CTAB protocol (Doyle & Doyle, 1987). The AFLP procedure was performed following the established protocols (Vos et al., 1995; PE Applied Biosystems). Genomic DNA (≈0.5 μg) was digested with two restriction endonucleases, EcoRI and MseI, and ligated to double‐stranded EcoRI and MseI adaptors. Preselective and selective amplifications were performed as described by Vos et al. (1995). The final three primer combinations for selective PCR were EcoRI (Fam)‐ACC/MseI‐CAT, EcoRI (Hex)‐ACG/MseI CAG, and EcoRI (Ned)‐AGC/MseI‐CAG. The three combinations of AFLP primers generated 203 unambiguous AFLP bands (hereafter referred to as bands) (EcoRI [Ned]‐AGC/Mse ICAG:90 fragments; EcoRI [Hex]‐ACG/Mse I‐CAG:90; Eco RI[Fam]‐ACC/ Mse I‐CAT:23). All but one were polymorphic within each of the 11 populations investigated, which allowed us to distinguish unique multilocus phenotypes for each of the 255 trees analyzed (for more details, see Terrab et al., 2008). Details of the number of fragments, the percentage of polymorphic fragments, and the number of private fragments per population are provided in Table 1.

**TABLE 1:** Locations of *Cedrus atlantica*.

Pop.	Location	Region	Coordinates	No. ind.	Frag_tot_	Frag_poly_	Frag_priv_	Genetic cluster	Nei's GD	DW
1	Jbel Bou‐Hachem	Rif	35°14′ N 5°25′ W	24	112	67.85	3	1	0.104	15.137
2	Ketama	Rif	34°58′ N 4°40′ W	23	113	76.10	3	1	**0.110**	18.539
3	Tazzeka	Middle Atlas	34°05′ N 4°10′ W	26	108	75.92	0	1	0.104	13.730
4	Azrou	Middle Atlas	33°25′ N 5°13′ W	24	106	83.01	0	1	**0.114**	13.755
5	Col de Zad	Middle Atlas	33°00′ N 5°04′ W	25	107	67.28	0	1	0.095	13.908
6	Ifran	Middle Atlas	33°37′ N 5°03′ W	17	145	90.34	1	2	**0.203**	22.373
7	Aguelmam Aziza	Middle Atlas	32°58′ N 5°26′ W	18	134	73.88	2	2	0.152	19.945
8	Jbel Bou‐Iblan	Middle Atlas	33°38′ N 4°13′ W	23	149	75.83	0	2	0.157	21.374
9	Theniet el Had	Tell Atlas	35°52′ N 1°56′ E	24	140	82.85	5	2	**0.179**	24.701
10	Djurjura: Tikjda	Tell Atlas	36°26′ N 4°07′ E	24	122	73.77	8	3	**0.105**	20.193
11	Jbel Cheliah	Aurès mountains	35°18′ N 6°37′ E	27	125	79.20	2	3	**0.132**	18.345

*Note*: AFLP diversity and rarity descriptors. Bold indicates the two highest diversity values within the genetic groups and, therefore, the greatest interest for conservation according to the calculation of the RGUCs.

Abbreviations: AFLP, amplified fragment length polymorphism; DW, frequency down‐weighted marker values; Nei's GD, Nei's diversity index (Nei, 1987); RGUCs, relevant genetic units for conservation.

### 2.2 Selection of RGUCs

Since the analysis requires the establishment of the genetic structure of the species, the isolation by distance (IBD) pattern was explored. Mantel's test (Mantel, 1967) was performed using the software ALLELES IN SPACE (AIS; Miller, 2005). The regression of pairwise genetic distance values was calculated (measured as the proportion of mismatched loci) against geographic distances (at a logarithmic scale) between pairs of individuals. We used AIS to perform a spatial autocorrelation analysis (SAA) to examine the correlation between genetic and geographic distances in multiple distance classes. Autocorrelation was measured using the parameter A_y_, which quantifies the average genetic distance between pairs of individuals that fall into different distance classes (Miller, 2005). A total of 1000 replicates were used to calculate the significance of each distance class. The IBD pattern was excluded, given the lack of correlation between genetic and geographic distances (Figures S1 and S2). Subsequently, using the spatial model option of the Bayesian analysis of population structure software (BAPS v. 6.0; Corander et al., 2008; Corander & Marttinen, 2006), we determined the genetic population structure of the species. This analysis clustered the samples into probable numbers of genetic groups using a Markov chain Monte Carlo simulation. We tested for a different number of groups, from one (all populations belonging to the same group) to 11 (all populations are different from each other and each belongs to a different group).

Relevant genetic units for conservation selection relies on population structure and the probability of rare allele loss (Caujapé‐Castells & Pedrola‐Monfort, 2004; Pérez‐Collazos et al., 2008). To calculate the number of populations (*n*) that should be preserved to represent a known proportion of genetic diversity (*P*), we solved the equation *P* = 1 − *F*_ST_^*n*^ (Segarra‐Moragues & Catalán, 2010). *F*_ST_, *F*‐statistic, is a measure of population differentiation due to genetic structure, which was calculated using ARLEQUIN 3.5.1.2 (Excoffier & Lischer, 2010). The conservation target (*P*) for *C. atlantica* populations was set at 99% of the total genetic diversity.

**FIGURE 2:**
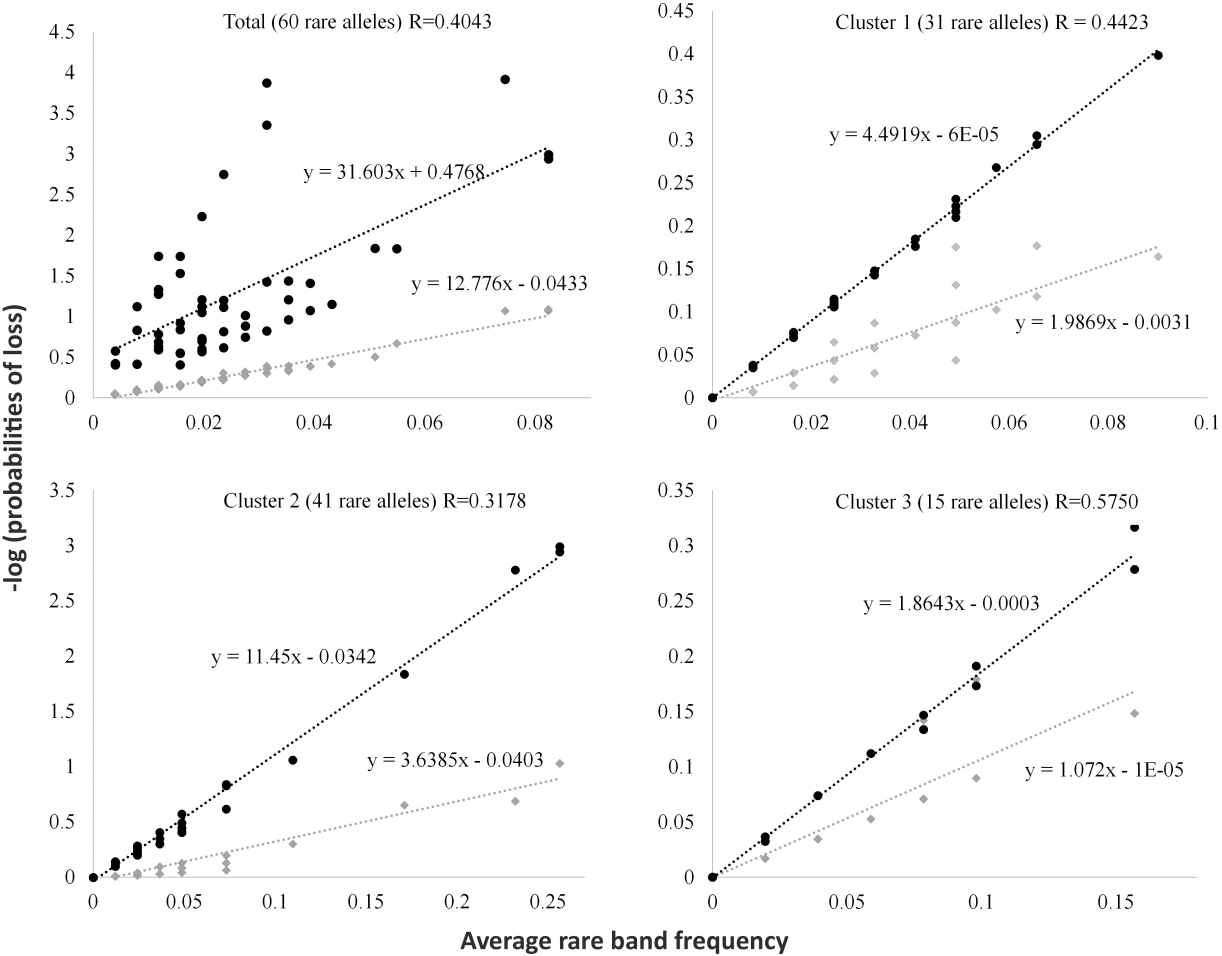
Regression lines of the average rare bands frequency (*x*‐axis) with the negative logarithms of the observed and expected probabilities of loss (−log[L_o_] [gray diamonds] and −log[L_e_] [black circles]) over the full set of rare amplified fragment length polymorphism bands and over the genetic clusters (C1, C2, and C3) of *Cedrus atlantica*. The quotient between the slopes of the observed and the expected regression lines indicates the percentage of rare AFLP bands represented when sampling a single population within the clusters (*R*‐value).

Furthermore, we defined rare alleles as those with an overall frequency lower than 10% and existing in less than 40% of the population (Table 2). These were used rather than the pre‐established values (10%–10% or 10%–20%; Peñas et al., 2016; Pérez‐Collazos et al., 2008) due to the relatively low number of rare AFLP alleles obtained with the other values (Caujapé‐Castells & Pedrola‐Monfort, 2004). Regarding the probability of loss of rare bands, we used the equation *L* = (1 − *p*)^2*N*^ (Bengtsson et al., 1995) which considers the band frequency of each rare allele (*p*) and the number of populations in which the rare band is present (*N*) (Pérez‐Collazos et al., 2008). For each rare band, the observed and expected probabilities of loss (*L*_o_ and *L*_e_, respectively) were calculated using the method described by Pérez‐Collazos et al. (2008). It was then possible to calculate the *R*‐value, which is the proportion of rare alleles (bands in this case) captured by sampling only one population (Bengtsson et al., 1995; Caujapé‐Castells & Pedrola‐Monfort, 2004; Pérez‐Collazos et al., 2008; Segarra‐Moragues & Catalán, 2010). The *R*‐value is calculated as the quotient between the slope of the expected regression line and the slope of the observed regression line of the negative natural logarithm of the probabilities of loss of each rare band (*R* = m[−log *L*_o_]/m[−log *L*_e_]; Figure 2). *R*‐values were calculated for the species as a whole and the genetic clusters revealed by BAPS.

**TABLE 2:** Rare AFLP bands distribution (those with an overall frequency lower than 10%, and present in <40% of the populations) and RGUCs values obtained for the full range of *Cedrus atlantica* and for the three genetic clusters (C1, C2, and C3).

	Full range	C1	C2	C3
Total no. AFLP bands	203	151	181	142
No. rare bands	60	31	41	15
% of rare bands	29.56	15.27	20.20	7.39
No. rare bands (by PSA)	—	27	20	13
% of rare bands (by PSA)	—	45.00	33.33	21.67
No. of exclusive bands	—	11	16	8
*R*‐value (%)	40.43	44.23	31.78	57.50
Optimal proportion	—	0.38	0.28	0.34
*n*	4.94	1.88	1.37	1.67
*n* (integer)	—	2	2	2

*Note*: *R*‐value: percentage of rare AFLP bands captured by sampling one population within the genetic clusters; *n*: calculated number of populations to be sampled to include a fixed diversity value, that is, 99%.

Abbreviations: AFLP, amplified fragment length polymorphism; PSA, preferred sampling area; RGUCs, relevant genetic units for conservation.

To determine which genetic clusters have a higher probability of gathering rare bands, we calculated the preferred sampling areas (PSA; preferred sampling clusters in our case) for each rare band. We took into account the percentage of the population in which a rare band was present within the genetic clusters and the number of individuals within the clusters in which a rare band was present. The optimal proportion of the populations sampled in each cluster was calculated based on the PSA percentages and *R*‐values of the genetic clusters. For each PSA, the best populations to be considered as RGUC were selected by considering the higher value of Nei's gene diversity index (Nei, 1987) as calculated by R (v.3.6.3.) script AFLPdat (Ehrich, 2006). Moreover, the frequency down‐weighted marker values (DW; Schönswetter & Tribsch, [2005]) were calculated to reinforce this decision. To check the validity of the PSA proposal, we calculated the number of AFLP bands (both rare and not rare) that would be captured if the population selection process had been random. The average values were calculated over 100 repetitions. Finally, the values of rarity and genetic diversity were represented on a map using the Multilevel b‐spline tool (Conrad et al., 2015) implemented in QGIS v.3.16.2 (QGIS‐Development‐Team, 2017) to display the general patterns of rarity and diversity of the species.

### 2.3 Designing the seed bank

To design an optimal seed bank, we calculated the relative contribution of the populations to the genetic pool that maximized the species' genetic diversity using the software Metapop2 v2.2.1 (López‐Cortegano et al., 2019). This software calculated the expected proportional contribution to a theoretical synthetic pool with maximum gene diversity (*D*_max_). The expected contributions were calculated for BAPS genetic clusters using all populations as a whole. This was done by maximizing the function $D_{\max}=1-\sum_{\mathrm{ij}=1}^{n}f_{\mathrm{ij}}\;c_{i}\;c_{j}$, where f_*ij*_ is the average coancestry between populations *i* and *j* and c_*i*_ is the contribution of subpopulation *i* to the pool (Toro & Caballero, 2005). A total of 100 replicates and a synthetic pool of 1000 individuals were used. The software also calculates the proportional contribution of each population to Nei's gene diversity (Δ*H*_nei_; Nei, 1978) and the proportional contribution of each population to the average Nei's minimum genetic distance (Δ*H*_dist_) between populations to interpret the importance of the populations in the seed bank. To evaluate the efficiency of selection, the software calculated the genetic diversity of a randomly selected seed bank using the same parameters.

### 2.4 Modeling genetic structure and spatial prediction

To characterize current climatic conditions (1970–2000), we obtained 11 out of the 19 bioclimatic variables from the WorldClim dataset v2.1 (www.worldclim.org): Annual Mean Temperature (Bio1), Mean Diurnal Range (Bio2), Isothermality (Bio3), Temperature Seasonality (Bio4), Maximum Temperature of Warmest Month (Bio5), Minimum Temperature of Coldest Month (Bio6), Temperature Annual Range (Bio7), Annual Precipitation (Bio12), Precipitation of Wettest Month (Bio13), Precipitation of Driest Month (Bio14), and Precipitation Seasonality (Bio15). We used these variables since they describe different aspects of temperature and precipitation regimes, discarding quarterly variables (mean temperature of wettest quarter, mean temperature of driest quarter, mean temperature of warmest quarter, mean temperature of coldest quarter, precipitation of wettest quarter, precipitation of driest quarter, precipitation of warmest quarter, and precipitation of the coldest quarter). These quarterly variables were discarded because they correlated with monthly variables in the study area or because they combined temperature and precipitation regimes, hampered the interpretation of the results, and created meaningless patterns in regions with high seasonality in temperature and precipitation. We downloaded the data at a 2.5 arcmin resolution (~5 km^2^ at the equator), which were cropped to the study area defined by a bounding box from −9° W to 7° E and from 30° N to 38° N. To avoid multicollinearity problems, the initial 11 bioclimatic variables were analyzed using a variance inflation factor analysis, using 0.7 as the *R* ^2^ threshold (Dormann et al., 2012). This analysis correlated each variable with the others, discarding those that had a high multivariate correlation with other variables in the data set, resulting in a final subset of five variables being included in the subsequent analysis: Bio1, 3, 4, 13, and 14. Since no IBD signal was identified in the genetic analysis, no spatial variables were included in the models.

To study the relationship between genetic composition and climatic variables and make spatially explicit projections, we used Gradient Forest (GF), as implemented in the gradientForest package v0.1–32 for *R* (Ellis et al., 2012). GF is a multivariate extension of Random Forest (RF), in which an RF model is fitted to relate each locus to multiple predictor variables (such as bioclimatic variables). Subsequently, all individual models are combined to identify (a) points along environmental gradients maximizing the changes in genetic composition and (b) the predictor variables with the greatest contribution to explaining the overall genetic turnover. Although GF might be more limited in identifying local adaptations than other techniques (BayeScan or BayeScEnv; Fischer et al., 2011; Fitzpatrick & Keller, 2015; Villemereuil & Gaggiotti, 2015), it allows relating the whole genetic data to multiple independent variables simultaneously. This is relevant in our study because of the nature of the genetic data (such as AFLP bands instead of alleles). The low number of bands prevented strict analysis of local adaptations to specific variables. Furthermore, genetic patterns might emerge from multiple alleles co‐varying in a similar or contrasting manner with multiple variables simultaneously (Ellis et al., 2012; Fitzpatrick & Keller, 2015). In our analysis, we fitted the GF for all bands in the AFLP data set using a classification algorithm (dependent variables were the presence or absence of specific alleles in each band) and 500 trees in each RF model.

Gradient forest allows the estimation of patterns of turnover in biological composition using nonlinear functions (from RF models) of each environmental gradient. Therefore, it performs biologically informed transformations of bioclimatic variables into genetic importance values (Fitzpatrick & Keller, 2015). These genetic importance values are the result of projecting the model to environmental and geographical spaces, allowing the identification of areas with similar predicted genetic structures, and were the basis for the final interpretation and representation. First, the values for each *C. atlantica* population were analyzed using hierarchical cluster analysis. To test whether climate can explain the observed genetic structure, we expected to observe three groups in the genetic importance variables. Next, to plot the results, we analyzed their values with principal component analysis (PCA), in which eigenvalues were used to rotate the genetic importance values for the remaining study area. The first three axes of the rotated variables were plotted as biplots and maps, illustrating their positions in the environmental and geographic spaces. This procedure was performed with current and future climate projections from 24 climate models in the worst‐case scenario (RCP8.5) available in the WorldClim database, whose results were then averaged (Figure S3).

## 3 RESULTS

### 3.1 Selection of RGUCs

From 203 AFLP bands, 60 met the rarity requirements (Table 2). BAPS cluster analysis resulted in an optimal partition of *K* = 3 clusters (Figure 1). Cluster 1 included populations 1–5. Cluster 2, which had the highest admixture degree, included populations 6–9, whereas Cluster 3 included populations 10 and 11. Given this genetic structure, the distribution of the rare AFLP bands was as follows: 11 rare AFLP bands were exclusive to Cluster 1, 16 were exclusive to Cluster 2, and eight were exclusive to Cluster 3. After choosing the PSAs for each of the rare bands (Table 2 and Table S1), 27 were assigned to Cluster 1, 20 to Cluster 2, and 13 to Cluster 3. The number of rare bands captured when sampling randomly only one population per region (*R*‐value; Figure 2) ranged from 31.78% for Cluster 2 to 57.50% for Cluster 3. Cluster 1 had an intermediate *R*‐value (44.23%) with a similar value (40.43%) estimated when considering the entire range of the species. Considering the PSA distribution and the *R*‐values of the different clusters, the optimal proportions of the populations to be sampled for each cluster were 0.38 (Cluster 1), 0.28 (Cluster 2), and 0.34 (Cluster 3). Given the *F*_ST_ calculated value of 0.247 and a target of 99.9% of the diversity, the optimal number of populations to be sampled was 4.94. Applying this method to the different genetic clusters, the optimal number of populations to be sampled for all three clusters was two: Cluster 1 (1.88), Cluster 2 (1.37), and Cluster 3 (1.67). Values were rounded up to avoid underestimating the conservation goals.

**FIGURE 3:**
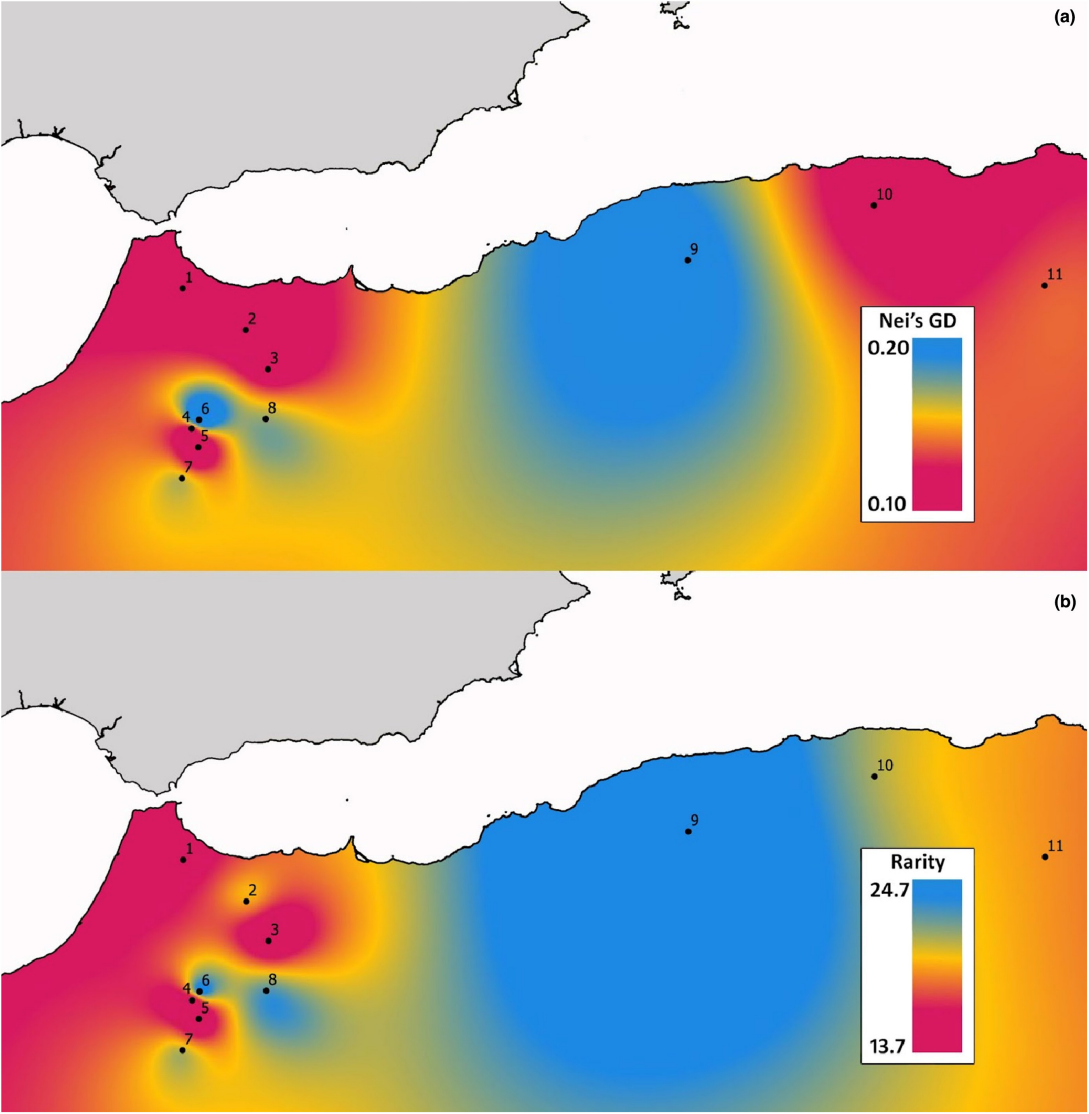
(a) Nei's gene diversity and (b) rarity patterns (red = low; orange = medium; blue = high) calculated with the multilevel b‐spline interpolation tool implemented by QGIS.

Nei's diversity values of the populations ranged from 0.095 in population 5 (Cluster 1) to 0.203 in population 6 (Cluster 2). The diversity values presented a general pattern of low genetic diversity. Populations 6 and 8 from the Middle Atlas and population 9 from the Tell Atlas had relatively higher levels of genetic diversity (Figure 3). Rarity ranged from 13.7 in population 3 (Cluster 1) to 24.7 in population 9 (Figure 3 and Table 1), following a similar pattern to the diversity values. Populations 6 and 8 from the Middle Atlas and population 9 from the Tell Atlas presented relatively higher levels of rarity. Within the genetic clusters, the populations with higher values within Cluster 1 were populations 2 and 4, with diversity values of 0.110 and 0.114, respectively, and populations 1 and 2 with rarity values of 15.1% and 18.5%, respectively. For Cluster 2, populations 6 and 9 showed the highest diversity and rarity values of 0.203, 0.179, 22.4, and 24.7, respectively. Lastly, only two populations of Cluster 3 (populations 10 and 11) showed genetic diversities of 0.105 and 0.132, respectively, with a population rarity of 20.2% and 18.3%. The selection as RGUCs of the two populations per cluster with higher values of Nei's diversity gathered 97.53% of the total AFLP bands and 90% for the rare AFLP bands. The randomization resulted in a gathering of 93.39% of total AFLP bands, while for rare AFLP bands it was 79.3%.

### 3.2 Population contribution to the seed bank

The optimal contribution of the populations that maximized the genetic diversity (Table 3), considering the species as a whole, was gathered when the seed bank was composed of 26.1% of population 2, 56.8% of population 6, and 17.1% of population 9. However, when considering the three genetic clusters separately, the values for Cluster 1 were 14.1, 34.3, and 51.6 for populations 1, 2, and 4, respectively. For Cluster 2, they were 63.7 and 36.3% for populations 6 and 9, respectively. For Cluster 3, they were 25.8 and 74.2% for populations 10 and 11, respectively. Given the decrease in genetic diversity and genetic distance when the populations were disregarded one by one, population 4 in Cluster 1 exhibited the highest reduction in genetic diversity and genetic distance values (1.84% and 1.21%, respectively). In Cluster 2, the highest decrease in genetic diversity was found in population 6 (4.16%), whereas the highest decrease in the genetic distance was in population 9 (3.34%). In Cluster 3, the highest decrease in genetic diversity and genetic distance was found in population 11 (13.4% and 13.05%, respectively), although population 10 also showed a high decrease in the genetic distance (10.41%). The values of Nei's genetic diversity calculated for the three synthetic populations were 0.17, 0.224, and 0.172 (for Clusters 1, 2, and 3, respectively), representing an increase of 53%, 22.1%, and 35.7% regarding the random selection of the populations and percentages in the creation of the seed bank. In addition, Nei's diversity values of the randomly created synthetic populations were 0.111, 0.183, and 0.126 for Clusters 1, 2, and 3, respectively.

**TABLE 3:** Results obtained with Metapop2 v2.2.1

Population	Cluster	% Total	%/Cluster	∆*H*_nei_ (%)	∆*H*_dist_ (%)	∆*H*_t_ (%)
1	1	0	14.1	−0.2068	0.8043	0.5975
2		26.1	34.3	0.8594	0.4164	1.2759
3		0	0	−0.1388	−0.1343	−0.2731
4		0	51.6	1.8441	1.2119	3.056
5		0	0	−2.2771	0.0677	−2.2094
6	2	56.8	63.7	4.1927	1.7751	5.9678
7		0	0	−2.9799	0.0805	−2.8994
8		0	0	−2.6957	0.4314	−2.2643
9		17.1	36.3	1.8492	3.3439	5.1931
10	3	0	25.8	−9.5216	10.4141	0.8925
11		0	74.2	13.429	13.0558	26.4848

*Note*: Δ*H*_nei_, proportional variation of the within‐population gene diversity when the population data are removed in the analysis; Δ*H*_dist_, proportional variation of Nei's average genetic distance between populations when the population data are removed in the analysis; Δ*H*_t_, total variation; Δ*H*_nei_, Δ*H*_dist_, and Δ*H*_t_ are referred to cluster analysis; for the variations, positive values showing that the indicator decreases by removing mentioned populations, and negative value showing that the indicator increases by removing mentioned populations; % total, expected proportion of seeds from the populations in order to obtain the maximum diversity values in a synthetic population considering all the populations; %/cluster, expected proportion of seeds from the populations in order to obtain the maximum diversity values in a synthetic population within each genetic cluster.

### 3.3 Gradient forest and spatial genetic patterns

**FIGURE 4:**
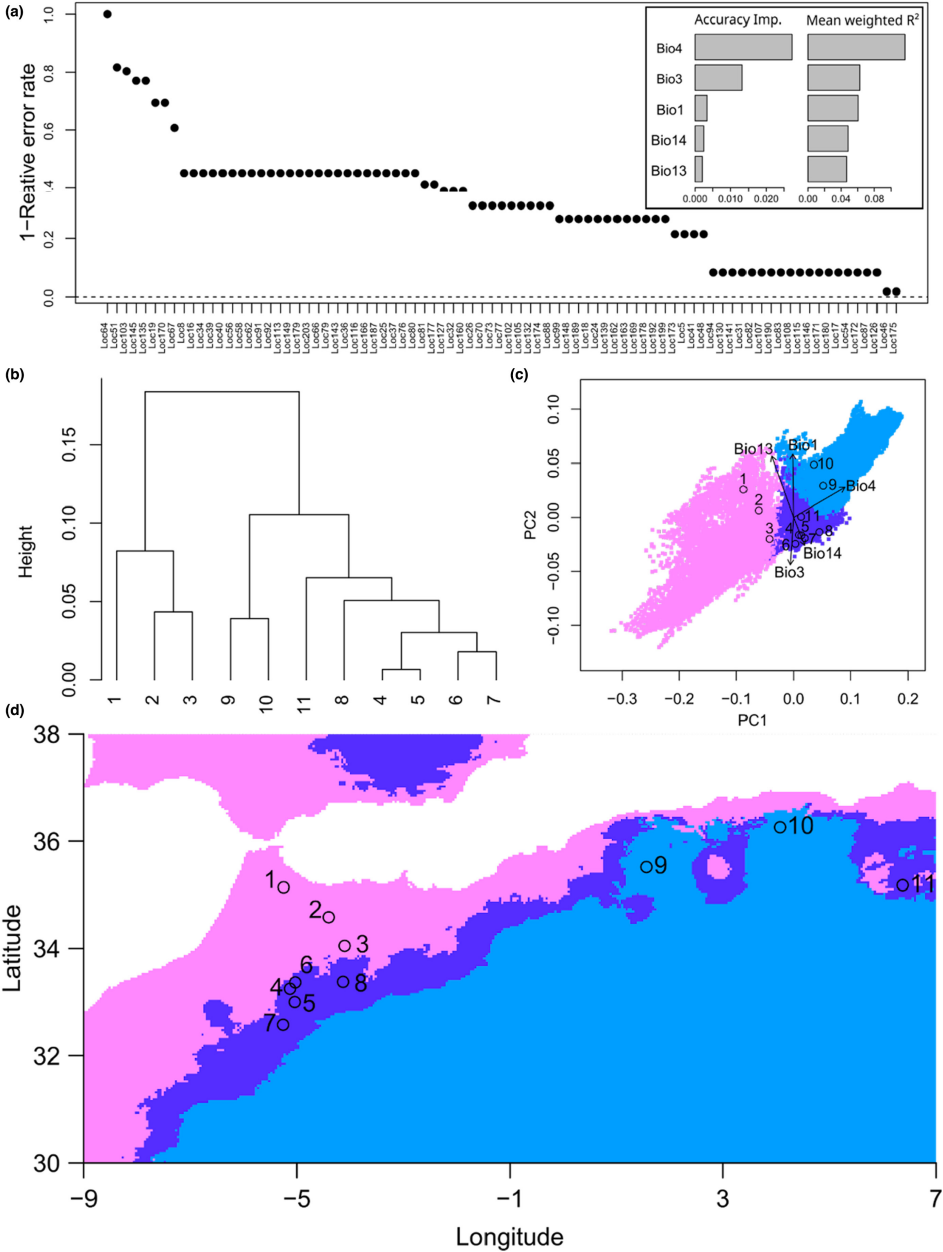
Relationship between *Cedrus atlantica* genetic composition and environmental variables as estimated from the gradient forest model. (a) Model accuracy estimating locus composition and importance of environmental variables through two metrics (Accuracy importance and Mean Weighted *R* ^2^), (b) cluster analysis of *C. atlantica* populations based on the projection of the gradient forest model, (c) principal component analysis (PCA) of projection values of the gradient forest model for the whole study area (colored squares) and *C. atlantica* populations (black circles) with eigenvalues of environmental variables for the two first axis of the PCA analysis, and (d) projection of gradient forest model to the geographic space of the whole study area. The map was performed by running a Principal Component Analysis on the GF projections and using the scores from the three first axes in a cluster analysis to match each pixel to one of the three groups in ‘b’. Areas with the same color represent areas with environmental conditions expected to host populations with similar genetic composition.

The gradient forest model found a relationship between allele composition for 86 out of the 203 AFLP bands, although only 10 bands could be modeled with error rates of 0.5 or lower (Figure 4a). The most significant variables explaining the genetic composition of the *C. atlantica* populations (Figure 4a) were temperature seasonality (Bio4), annual temperature range (Bio7), isothermality (Bio3), and maximum temperature of the warmest month (Bio5). Hierarchical cluster analysis of the population values of the transformed bioclimatic variables by GF (Figure 4b) identified three population groups: Group 1 with populations 1–3; Group 2 with populations 9 and 10; and Group 3 with populations 4–8 and 11. However, ordering the populations in the biplot from the PCA of the values placed population 11 in an intermediate position between the three groups (Figure 4c and Figure S4). The spatial projection of the PCA rotated variables suggested that the Atlantic and Mediterranean coastal areas in North Africa have climatic conditions favoring a genetic composition similar to that of Group 1 (Figure 4d and Figure S4). Group 2 would be favored in inner locations close to the Sahara (Figure 4d and Figure S4), whereas Group 3 would be favored in intermediate areas along the Atlas Mountain (Figure 4d and Figure S4). Population 11 was located in an area with climatic conditions more similar to Group 3 but was closely placed to populations 9 and 10 spatially (Figures 1, 3, and 4d). Model projection of future climate conditions resulted in a predominance of climate conditions favoring genetic structures similar to those in Group 2. All studied populations, except number 1, were expected to be under such conditions (Figure S3).

## 4 DISCUSSION

### 4.1 RGUCs as in situ conservation proposal

The conservation of tree species in their original habitats, especially in mountainous areas, is the primary objective of conservation biologists, as these areas function as refugia and have higher ecological stability (Hewitt, 2004; Tzedakis et al., 2002).

Although all populations of threatened species should receive in situ conservation attention, this is rarely possible for economic and logistical reasons. Considering *C. atlantica*, conservation would be more efficient in the long term if it focused on the most suitable areas and populations with specific adaptations (Cheddadi et al., 2022). The highest priority for in situ conservation is to capture as much genetic variability as possible (Falk & Holsinger, 1991). Therefore, we must consider the diversity and rarity of all population species (Ciofi & Bruford, 1999; Ryder, 1986). AFLP data have been used to analyze genetic diversity in various threatened or rare gymnosperm taxa, such as *Picea ziyuanensis* (Tang et al., 2008), *Araucaria species* (Gaudeul et al., 2012), *Abies chensiensis*, *A. fargesii* (Zhan et al., 2014), and *Pinus monticola* (Kim et al., 2011), among others. The RGUCs sampling strategy allows us to determine which and how many populations to choose for conservation purposes (Peñas et al., 2016; Pérez‐Collazos et al., 2008). Plant genetic diversity is spatially structured at different scales such as geographical areas, populations, or neighboring individuals (Engelhardt et al., 2014) due to environmental influences, life‐history traits, and the demographic history of the species. Previous research has shown that *C. atlantica* has no clear geographical diversity structure (Terrab et al., 2006), and populations of each mountain range may have been isolated from each other for a long time (Renau‐Morata et al., 2005; Terrab et al., 2006). The lack of a clear spatial genetic structure within each mountain range could indicate a moderate genetic flow between subpopulations. Alternatively, it could indicate the relative success of one genotype compared with others that went extinct under adverse climatic conditions.

Regarding the BAPS results, *C. atlantica* was structured into three genetic clusters considered independent for conservation reasons. This result is similar to that obtained by Terrab et al. (2008) through NMDS, neighbor‐joining analysis, and Bayesian analysis. Cluster 1 included populations 1–5, Cluster 2 populations 6–9, and Cluster 3 populations 10 and 11. Populations 2 and 4 from Clusters 1, 6, and 9 from Cluster 2, and both populations from Cluster 3 were finally selected to cope with the in situ conservation goal as they represented the higher diversity values. This selection included 97.53% of the total AFLP bands and 90% of the rare AFLP bands, preserving only 54% of the population. Terrab et al. (2008) proposed that all populations studied deserve some attention in genetic conservation programs (only populations from Morocco were considered in this study). The authors also highlighted that the populations conserved were those corresponding to populations 2 and 4–6. This is the first time that a full range of *C. atlantica* has been considered for conservation measures. Although all populations should be preserved, the high level of AFLP bands included in the RGUCs makes conservation procedures reliable and efficient. In our case, the values of preserved rare bands were similar to those of RGUCs proposed in other species, such as *Androcymbium gramineum* (Cav.) McBride (Caujapé‐Castells & Pedrola‐Monfort, 2004), of which eight out of 13 populations were selected, gathering 97% of rare alleles. For *Boleum asperum*, four out of eight populations were selected (Pérez‐Collazos et al., 2008), which included 85.10% of the rare bands. Also, in *Borderea pyrenaica* Míegeville, 5 out of the 11 populations studied were selected (Segarra‐Moragues & Catalán, 2010), containing 97.5% of the rare alleles. The value obtained for *C. atlantica* was higher than in other species such as *Convolvulus boissieri* Steud., where six out of the 15 populations studied were selected around 61% of the rare fragments were preserved (Mota et al., 2019). For *Jacobaea auricula*, only 50% of the rare bands were selected for protection using the RGUCs selection (Bobo‐Pinilla et al., 2021).

### 4.2 Genetic contribution to seed bank design

The designation of protected areas is insufficient for protecting biodiversity (Volis, 2019; and references within). Conservation actions focused on passive protection may not prevent diversity loss (Fenu et al., 2019). A method that unifies the different aspects regarding the viability of populations as part of conservation proposals is necessary, which is deficient for ex situ conservation (De Rogatis et al., 2022; Volis & Blecher, 2010). Some authors have suggested ex situ conservation of *C. atlantica* (Cheddadi et al., 2022; Terrab et al., 2008). The populations necessary for an optimal seed bank were 1, 2, and 4 for Clusters 1; 6, and 9 for Cluster 2; and 10, and 11 for Cluster 3. This selection partially coincided with that obtained by the selection of RGUCs (populations 2 and 4 from Clusters 1; 6, and 9 from Cluster 2; and 10, and 11 from Cluster 3). However, the rarity value of population 1 makes it essential for ex situ conservation because rarity holds the adaptive potential of populations (Laikre et al., 2020), and conservation should be focused on the genetic distinctiveness of populations within a species (Bobo‐Pinilla et al., 2022; and references within). This proposal demonstrates the traditional problems of seed bank creation, where plant material was collected from several populations from different habitats, assuming that diversity was distributed along populations (Hamrick et al., 1991; Hamrick & Godt, 1990). The creation of an efficient seed bank that considers genetic diversity and rarity makes population reinforcements reliable and helps increase the success of conservation actions (Fenu et al., 2019; Lienert, 2004). The increase in the values of Nei's genetic diversity (53%, 22.1%, and 35.7% for Clusters 1, 2, and 3, respectively) regarding the random selection of seeds corroborates the need to establish specific studies for the creation of a seed bank.

### 4.3 Genetic spatial prediction models for conservation

Recently, Cheddadi et al. (2022) proposed the populations from Taffert (population 8) and Timghilt (not analyzed in this study but relatively close to population 3) as a potential source of seeds for reinforcements in the Moroccan populations, giving their high diversity values. However, effective population reinforcement would benefit from further identification of areas with high adaptive capacities for the genetic characteristics of seeds (Cheddadi et al., 2017).

The genetic grouping of the populations and their spatial projection to the whole study area based on the GF models provide spatially explicit guidance to choosing which of the three seed banks should be used. Therefore, bioclimatic variables with the highest influence on genetic composition were temperature‐related: temperature seasonality (Bio4), isothermality (Bio3), and mean annual temperature (Bio 1). Notably, these variables are consistent with several previous studies suggesting that climatic warming (Cheddadi et al., 2009; Linares et al., 2011; Mokrim, 2009) might cause *C. atlantica* to decline. Considering the genetic diversity values, reinforcement of the northernmost populations of Morocco (populations 1–3), all of which had low levels of genetic diversity, should be performed with the seed bank of the first genetic cluster. Populations 4–5, although they belong to genetic Cluster 1, are grouped with populations from genetic Cluster 2 (except for population 9) by the GF model. This could be because populations 4 and 5 have lost most of their original genetic diversity after reforestation programs or that these populations have survived in conditions for which they were not adapted (Navarro‐Cerrillo et al., 2013). The latter could explain the decline in these populations (Abel‐Schaad et al., 2018; González‐Hernández et al., 2022). Therefore, the proposal to reinforce these populations should focus on the climatic conditions of the area and the second genetic cluster seed bank should be used. Populations 6–8 from genetic Cluster 2 formed a homogeneous group from a climatic point of view (such as the GF projection). Their reinforcements could be performed with seeds from the same genetic cluster. The results were less clear in the Algerian population. Considering populations 10–11 (genetic Cluster 3), the GF model placed both populations in different clusters. This result suggests that both populations could be reinforced with different seed banks, but seed bank 3 would be made of 73% of population 11, which is for a climatic zone more likely supporting populations from genetic Cluster 2. Given the geographically isolated nature of these populations and the narrow climatic conditions for population 11, it appears reasonable to propose that these populations are reinforced with their seed banks. Alternatively, seed banks 2 and 3 should be tested to ensure that one has better adaptations to the climatic conditions in each population. Finally, the mismatches between the genetic grouping and the GF results, combined with their spatially isolated character and the climatic heterogeneity of the area (Figure 4 and Figure S4), could indicate a lack of equilibrium between these populations and their climatic conditions, which might explain the high rates of *C. atlantica* mortality reported in Algeria (Alileche et al., 2021; Kherchouche et al., 2013; Megdoud, 2012) and Morocco (El Abidine, 2003; Linares et al., 2011; Slimani et al., 2014).

Regarding the creation of new populations in climatically suitable areas, only a few very restricted areas located in the southwestern part of the High Atlas have been previously proposed as favorable for the survival of *C. atlantica* (Cheddadi et al., 2017; Terrab et al., 2008). Therefore, our approach makes a direct estimation to strengthen translocations. The ecological differences in the areas inhabited by plants make it probable that the introduction of individuals from different conditions decreases the survival and reproductive ability, as local adaptations could have developed within the areas (Fenster & Galloway, 2000; Lema & Nevitt, 2006). Moreover, several authors have proposed that gene flow among populations should be avoided to preserve their distinctiveness (Terrab et al., 2008). Although AFLP is a neutral marker and because the species is monoecious with a continuous variation in male/female allocation (such as quasi‐male to quasi‐female trees; Krouchi et al., 2004) and its mating system is mixed (trees produce a mixture of self‐fertilized and outbred seeds; Ferriol et al., 2011), the consideration of three genetic clusters to create a seed bank might reduce the risk of eliminating adaptation patterns as the transference of plant material within genetic areas reduces the risk of outbreeding (Kaulfuß & Reisch, 2017). Habitat fragmentation of *C. atlantica*, either natural or human‐induced, and the progressive reduction of some populations or maladaptation to new conditions (Figure S3) lead to a reduction in gene flow between populations and may increase the chances of its extinction (Cheddadi et al., 2017). Therefore, it is essential to have an efficient seed bank to limit the fragmentation effect on diversity and rarity. It also seems advisable to create migration corridors and/or establish “stepping stones” populations that could link current and future ranges (Hannah et al., 2007) or to provide assisted migration in which individuals are moved to new habitats of predicted suitability (Figure S3; Aitken et al., 2008; Rehfeldt & Jaquish, 2010).

## 5 CONCLUSIONS

Genetic research on *C. atlantica* regarding conservation, restoration, and exploitation is scarce. Here, we uncovered the existence of three relevant genetic units for the conservation of the species that maximized genetic diversity in a spatial context. Based on these RGUCs, their genetic diversity, and rarity, we propose a seed bank design, remarking the primary role of genetic markers for designing a reliable conservation strategy for *C. atlantica* forests considering current climate change, increased deforestation, and aridity in North Africa. Furthermore, we modeled genetic diversity as a function of climatic variables. Bioclimatic variables with the highest influence on genetic composition were highly related to climatic warming and decreased soil water availability. Projections of genetic composition due to the climate in the study region further provide spatially explicit guidance to use specific sources from the seed banks for *C. atlantica* population reinforcement and potentially for translocations. Although further research is required to validate this approach in the long term, we see it as an appropriate tool to inform conservation and management plans for forest species.

## AUTHOR CONTRIBUTIONS

**Javier Bobo‐Pinilla:** Conceptualization (equal); formal analysis (equal); investigation (equal); methodology (equal); software (equal); visualization (equal); writing – original draft (equal). **Diego Nieto Lugilde:** Conceptualization (equal); formal analysis (equal); investigation (equal); methodology (equal); software (equal); visualization (equal); writing – original draft (equal). **Anass Terrab:** Conceptualization (equal); supervision (equal); writing – review and editing (equal). **Francisco Balao:** Conceptualization (equal); supervision (equal); writing – review and editing (equal). **Julio Peñas:** Conceptualization (equal); formal analysis (equal); funding acquisition (equal); investigation (equal); project administration (equal); supervision (equal); writing – original draft (equal).

## CONFLICT OF INTEREST

The authors declare that they have no conflict of interest.

## Supporting information


Appendix S1

## Data Availability

Data available in article Supplementary Material; PSA details under Appendix S1, Table S1.
