# Supplementary material for: Spatially explicit assessment of genetic variation to inform conservation effort for an endangered Mediterranean conifer, Cedrus atlantica
Source: Ecol Evol. 2022 Dec 12;12(12):e9613. doi: 10.1002/ece3.9613 (PMC9745260; doi:10.1002/ece3.9613)
Supplement: Supplementary file 1 — Appendix S1 [file ECE3-12-e9613-s001.docx]

**SUPPLEMENTARY INFORMATION**

**TABLE S1.** Distribution and probabilities of loss of the 60 rare AFLP bands found in the *Cedrus atlantica* populations studied.

|  | **Cluster 1** | | **Cluster 2** | | **Cluster 3** | |  |  |  |  |
| --- | --- | --- | --- | --- | --- | --- | --- | --- | --- | --- |
|  | N inds. | N pops. | N inds. | N pops. | N inds. | N pops. | PSA | Lo | Le | Freq. indiv. |
| Loc102 | 2 | 2 | 6 | 2 | 0 | 0 | C2 | 0,40778 | 0.03729 | 0.03137 |
| Loc103 | 6 | 5 | 0 | 0 | 0 | 0 | C1 | 0.59619 | 0.24116 | 0.02353 |
| Loc108 | 0 | 0 | 4 | 2 | 0 | 0 | C2 | 0.70328 | 0.14428 | 0.01569 |
| Loc109 | 11 | 3 | 2 | 1 | 0 | 0 | C1 | 0.31524 | 0.01451 | 0.05098 |
| Loc11 | 3 | 3 | 0 | 0 | 0 | 0 | C1 | 0.76652 | 0.23168 | 0.01176 |
| Loc112 | 1 | 2 | 0 | 0 | 0 | 0 | C1 | 0.91493 | 0.37609 | 0.00392 |
| Loc113 | 0 | 0 | 0 | 0 | 8 | 1 | C3 | 0.49520 | 0.00044 | 0.03137 |
| Loc115 | 2 | 3 | 0 | 0 | 0 | 0 | C1 | 0.84028 | 0.38400 | 0.00784 |
| Loc117 | 3 | 3 | 1 | 1 | 0 | 0 | C1 | 0.70633 | 0.27949 | 0.01569 |
| Loc123 | 8 | 3 | 1 | 1 | 2 | 1 | C1 | 0.38095 | 0.07037 | 0.04314 |
| Loc124 | 3 | 4 | 1 | 1 | 0 | 0 | C1 | 0.71081 | 0.39113 | 0.01569 |
| Loc129 | 1 | 2 | 4 | 3 | 0 | 0 | C2 | 0.60335 | 0.24921 | 0.01961 |
| Loc132 | 0 | 0 | 19 | 3 | 0 | 0 | C2 | 0.08539 | 0.00012 | 0.07451 |
| Loc133 | 6 | 2 | 0 | 0 | 0 | 0 | C1 | 0.56250 | 0.00178 | 0.02353 |
| Loc137 | 3 | 2 | 2 | 1 | 0 | 0 | C1 | 0.60291 | 0.06186 | 0.01961 |
| Loc144 | 3 | 2 | 0 | 0 | 0 | 0 | C1 | 0.75614 | 0.04620 | 0.01176 |
| Loc145 | 4 | 4 | 0 | 0 | 0 | 0 | C1 | 0.70905 | 0.28345 | 0.01569 |
| Loc146 | 6 | 3 | 0 | 0 | 0 | 0 | C1 | 0.60546 | 0.06331 | 0.02353 |
| Loc149 | 0 | 0 | 0 | 0 | 5 | 1 | C3 | 0.62674 | 0.00586 | 0.01961 |
| Loc15 | 1 | 2 | 4 | 2 | 0 | 0 | C2 | 0.64427 | 0.19949 | 0.01961 |
| Loc151 | 2 | 2 | 1 | 1 | 0 | 0 | C1 | 0.74995 | 0.20543 | 0.01176 |
| Loc156 | 6 | 4 | 2 | 1 | 0 | 0 | C1 | 0.50171 | 0.15005 | 0.03137 |
| Loc16 | 0 | 0 | 0 | 0 | 2 | 1 | C3 | 0.84028 | 0.14745 | 0.00784 |
| Loc160 | 5 | 3 | 0 | 0 | 0 | 0 | C1 | 0.64403 | 0.08892 | 0.01961 |
| Loc161 | 3 | 2 | 0 | 0 | 0 | 0 | C1 | 0.75614 | 0.04620 | 0.01176 |
| Loc164 | 1 | 2 | 2 | 1 | 0 | 0 | C1 | 0.72006 | 0.16426 | 0.01176 |
| Loc17 | 4 | 3 | 1 | 1 | 0 | 0 | C1 | 0.63253 | 0.18648 | 0.01961 |
| Loc171 | 0 | 0 | 2 | 1 | 1 | 1 | C3 | 0.78013 | 0.25523 | 0.01176 |
| Loc172 | 6 | 3 | 1 | 1 | 0 | 0 | C1 | 0.52890 | 0.09676 | 0.02745 |
| Loc173 | 7 | 3 | 1 | 1 | 2 | 1 | C1 | 0.40647 | 0.08410 | 0.03922 |
| Loc174 | 0 | 0 | 6 | 3 | 0 | 0 | C2 | 0.49580 | 0.07635 | 0.02353 |
| Loc175 | 0 | 0 | 21 | 4 | 0 | 0 | C2 | 0.08524 | 0.00115 | 0.08235 |
| Loc179 | 0 | 0 | 2 | 1 | 0 | 0 | C2 | 0.84028 | 0.14745 | 0.00784 |
| Loc180 | 0 | 0 | 2 | 1 | 1 | 1 | C3 | 0.78013 | 0.25523 | 0.01176 |
| Loc186 | 0 | 0 | 1 | 1 | 0 | 0 | C2 | 0.91840 | 0.39207 | 0.00392 |
| Loc198 | 6 | 3 | 1 | 1 | 0 | 0 | C1 | 0.52845 | 0.09646 | 0.02745 |
| Loc200 | 0 | 0 | 21 | 4 | 0 | 0 | C2 | 0.08174 | 0.00102 | 0.08235 |
| Loc203 | 0 | 0 | 0 | 0 | 1 | 1 | C3 | 0.91840 | 0.39207 | 0.00392 |
| Loc26 | 0 | 0 | 4 | 1 | 5 | 2 | C3 | 0.46813 | 0.06185 | 0.03529 |
| Loc32 | 4 | 2 | 1 | 1 | 0 | 0 | C1 | 0.62430 | 0.07493 | 0.01961 |
| Loc34 | 0 | 0 | 3 | 1 | 0 | 0 | C2 | 0.76563 | 0.05299 | 0.01176 |
| Loc39 | 0 | 0 | 4 | 1 | 0 | 0 | C2 | 0.69444 | 0.01811 | 0.01569 |
| Loc40 | 0 | 0 | 1 | 1 | 0 | 0 | C2 | 0.91840 | 0.39207 | 0.00392 |
| Loc46 | 0 | 0 | 14 | 4 | 0 | 0 | C2 | 0.21509 | 0.01461 | 0.05490 |
| Loc54 | 0 | 0 | 2 | 2 | 4 | 1 | C3 | 0.59931 | 0.15301 | 0.02353 |
| Loc56 | 0 | 0 | 0 | 0 | 3 | 1 | C3 | 0.76563 | 0.05299 | 0.01176 |
| Loc58 | 0 | 0 | 0 | 0 | 4 | 1 | C3 | 0.72565 | 0.02938 | 0.01569 |
| Loc62 | 0 | 0 | 3 | 1 | 0 | 0 | C2 | 0.69444 | 0.01811 | 0.01176 |
| Loc63 | 0 | 0 | 1 | 1 | 0 | 0 | C2 | 0.88581 | 0.26349 | 0.00392 |
| Loc64 | 2 | 3 | 0 | 0 | 0 | 0 | C1 | 0.84028 | 0.38400 | 0.00784 |
| Loc67 | 3 | 3 | 4 | 2 | 0 | 0 | C1 | 0.47681 | 0.13045 | 0.02745 |
| Loc69 | 2 | 2 | 3 | 3 | 0 | 0 | C2 | 0.62144 | 0.27030 | 0.01961 |
| Loc73 | 0 | 0 | 6 | 1 | 4 | 2 | C3 | 0.41212 | 0.03876 | 0.03922 |
| Loc77 | 0 | 0 | 9 | 3 | 0 | 0 | C2 | 0.40463 | 0.03624 | 0.03529 |
| Loc8 | 0 | 0 | 2 | 1 | 0 | 0 | C2 | 0.79012 | 0.07493 | 0.00784 |
| Loc81 | 8 | 4 | 1 | 1 | 0 | 0 | C1 | 0.44733 | 0.10945 | 0.03529 |
| Loc83 | 0 | 0 | 4 | 2 | 0 | 0 | C2 | 0.68001 | 0.11990 | 0.01569 |
| Loc85 | 5 | 3 | 2 | 2 | 0 | 0 | C1 | 0.53508 | 0.17912 | 0.02745 |
| Loc91 | 0 | 0 | 0 | 0 | 2 | 1 | C3 | 0.84028 | 0.14745 | 0.00784 |
| Loc92 | 0 | 0 | 0 | 0 | 8 | 1 | C3 | 0.44444 | 0.00013 | 0.03137 |

**FIGURE S1.** Mantel tests: Graphical representation of the genetic distances versus geographic distances (at a logarithmic scale).

**
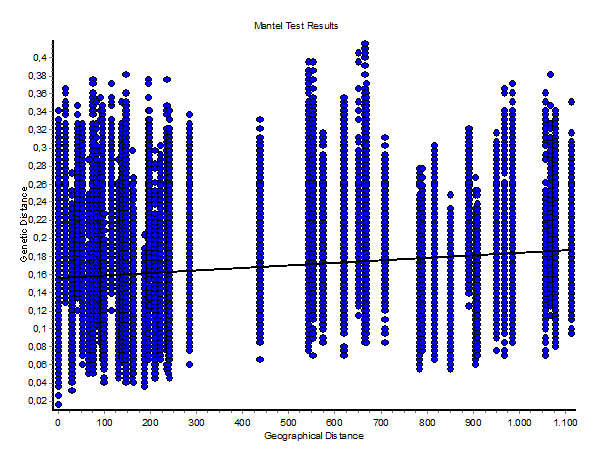
**

**FIGURE S2.** Results of spatial autocorrelation analyses. Ay quantifies the average pairwise genetic distances that fall within the distance class. Average value for Ay is represented by the horizontal dashed line.

**
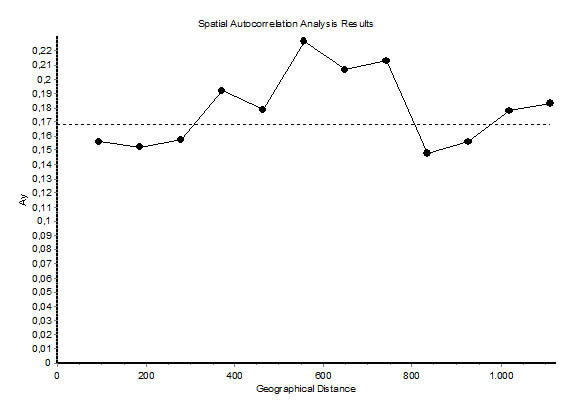
**

**FIGURE S3**. Gradient Forest projection of genetic composition under the worst scenario of climate change (i.e., RCP8.5). PCA of projection values of the Gradient Forest model for the whole study area and *C. atlantica* populations, as continuous (a) or clustered values (b), with eigenvalues of environmental variables for the two first axis of the PCA analysis. Projection of Gradient Forest model to the geographic space of the whole study area as continuous (a) and clustered values (b). The biplot and map of clustered values were performed by running a Principal Component Analysis on the GF projections and using the scores from the three first axes in a cluster analysis to match each pixel to one of populations in the three groups in Fig. S3b. Areas with same/similar colors represent areas with environmental conditions expected to host populations with similar genetic composition in the future.

**
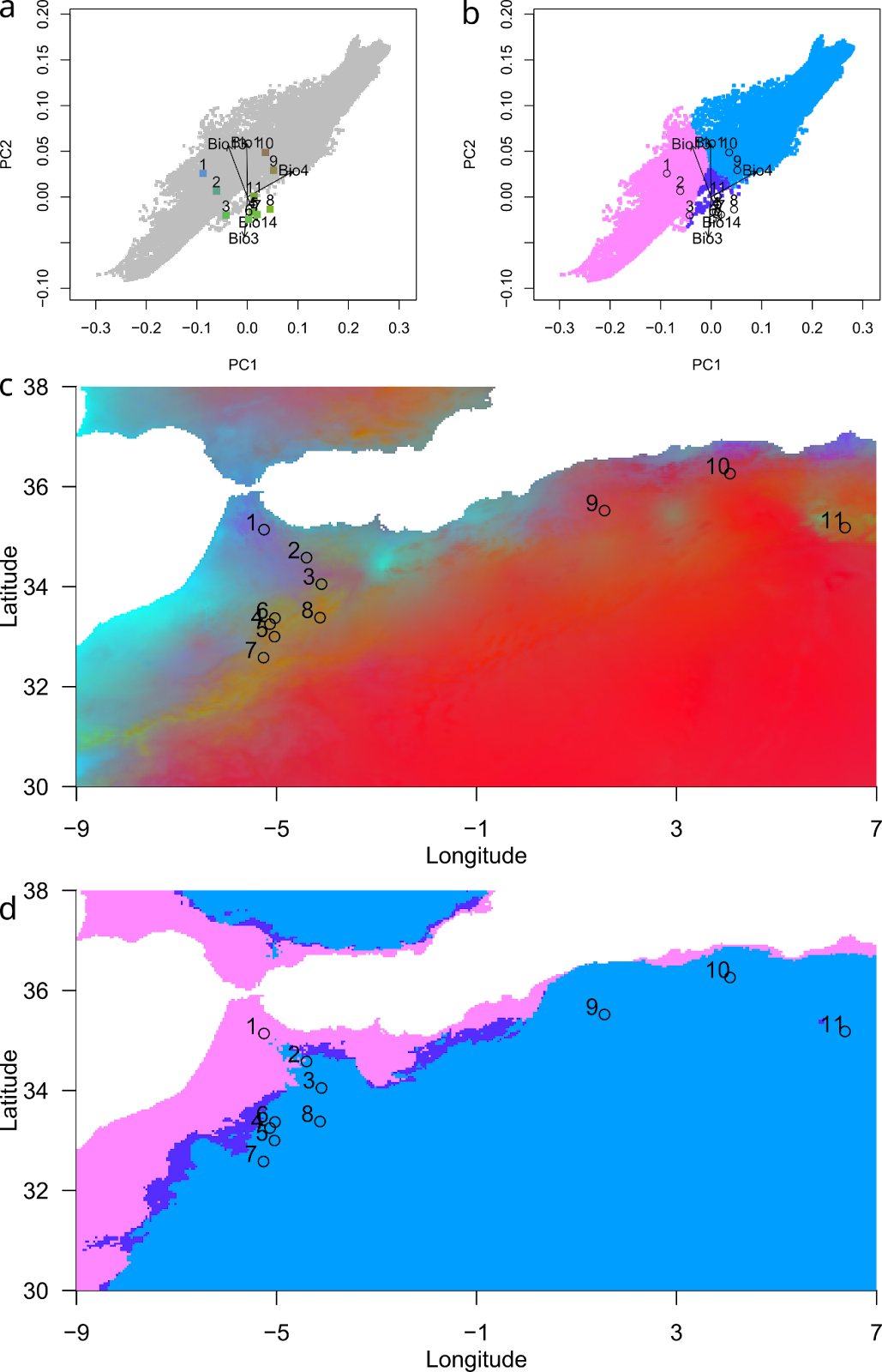
**

**FIGURE S4**. Relationship between *Cedrus atlantica* genetic composition and environmental variables as estimated from the Gradient Forest model. a) Model accuracy estimating locus composition and importance of environmental variables through two metrics (Accuracy importance and Mean Weigthed R^2^), b) cluster analysis of *C. atlantica* populations based on the projection of the Gradient Forest model, c) PCA of projection values of the Gradient Forest model for the whole study area (grey squares) and *C. atlantica* populations (colored squares) with eigenvalues of environmental variables for the two first axis of the PCA analysis, and d) projection of Gradient Forest model to the geographic space of the whole study area. The map was performed by running a Principal Component Analysis on the GF projections and using the scores from the three first axes as RGB composite colors, corresponding with colors in panel b. Areas with similar colors represent areas with environmental conditions expected to host populations with similar genetic composition.

**
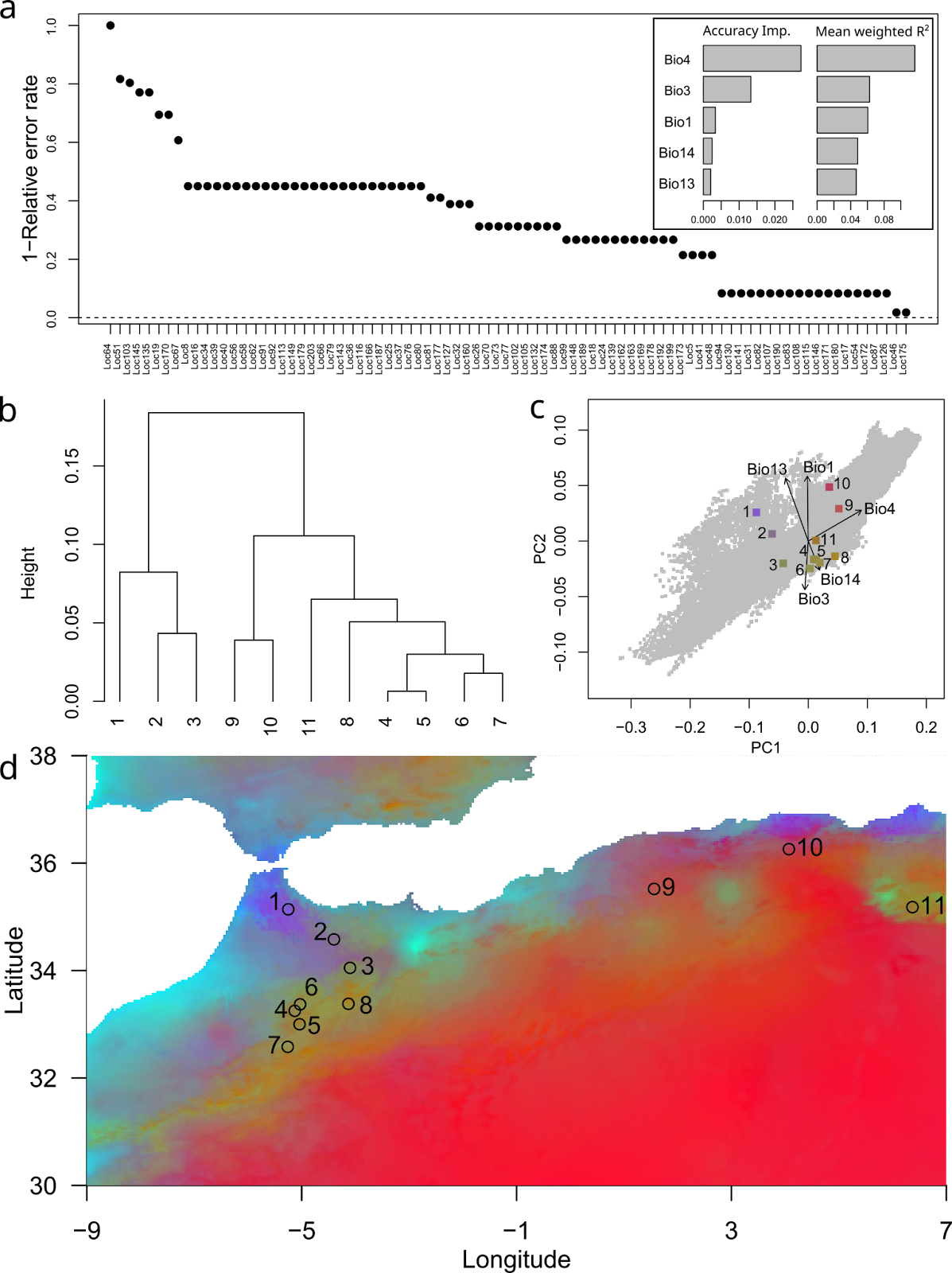
**
